# PPAR*γ* as a Novel Therapeutic Target in Lung Cancer

**DOI:** 10.1155/2016/8972570

**Published:** 2016-09-06

**Authors:** Aravind T. Reddy, Sowmya P. Lakshmi, Raju C. Reddy

**Affiliations:** ^1^Department of Medicine, Division of Pulmonary, Allergy and Critical Care Medicine, University of Pittsburgh School of Medicine, Pittsburgh, PA 15213, USA; ^2^Veterans Affairs Pittsburgh Healthcare System, Pittsburgh, PA 15240, USA

## Abstract

Lung cancer is the leading cause of cancer-related death, with more than half the patients having advanced-stage disease at the time of initial diagnosis and thus facing a poor prognosis. This dire situation poses a need for new approaches in prevention and treatment. Peroxisome proliferator-activated receptor *γ* (PPAR*γ*) is a ligand-activated transcription factor belonging to the nuclear hormone receptor superfamily. Its involvement in adipocyte differentiation and glucose and lipid homeostasis is well-recognized, but accumulating evidence now suggests that PPAR*γ* may also function as a tumor suppressor, inhibiting development of primary tumors and metastases in lung cancer and other malignancies. Besides having prodifferentiation, antiproliferative, and proapoptotic effects, PPAR*γ* agonists have been shown to prevent cancer cells from acquiring the migratory and invasive capabilities essential for successful metastasis. Angiogenesis and secretion of certain matrix metalloproteinases and extracellular matrix proteins within the tumor microenvironment are also regulated by PPAR*γ*. This review of the current literature highlights the potential of PPAR*γ* agonists as novel therapeutic modalities in lung cancer, either as monotherapy or in combination with standard cytotoxic chemotherapy.

## 1. Introduction

Lung cancer is the primary cause of cancer-related death, accounting for more than 25% of the total [1]. It falls into two major categories, small cell lung cancer (SCLC) and non-small cell lung cancer (NSCLC). NSCLC, comprising adenocarcinoma, squamous cell carcinoma, and large cell carcinoma, is more common, amounting to as much as 85% of all lung cancers, while SCLC represents the remaining 15% [2, 3]. Unlike many other cancers that have seen great improvement in 5-year survival during the last 3 decades, the 5-year relative survival of all patients with lung cancer remains below 18% [1, 4]. This is partially because more than half of lung cancers are diagnosed at an advanced stage [1], with 5-year survival of approximately 2% in this group [4], but also because progress in treatment has been slow [1]. Thus, new approaches to prevention, early detection, and treatment are urgently needed to enhance clinical outcomes.

Many tumors arise as a consequence of cells acquiring the ability to dedifferentiate or elude terminal differentiation, to proliferate in an unrestricted manner, and to escape apoptosis. Therapeutic intervention to reverse these processes thus remains an important strategy in cancer treatment. One approach that has attracted much interest is activation of nuclear hormone receptors, which has shown therapeutic potential in several major human diseases [5, 6]; agonists for estrogen receptor-*β*, retinoic acid receptor-*α*, and retinoid X receptor have demonstrated prodifferentiation, antiproliferative, and/or proapoptotic effects in a variety of cancers and cancer-related systems [7]. Another family of nuclear hormone receptors, the peroxisome proliferator-activated receptors (PPARs), has been long recognized for its role in regulation of lipid and glucose metabolism [8, 9]. It is now known that PPARs also regulate other cellular processes, among which are carcinogenesis-relevant cell differentiation, proliferation, survival, and apoptosis [10–12], with these effects being mediated via transcriptional activation or repression of PPAR target genes [9]. Three PPAR members, PPAR*α*, PPAR*β*/*δ*, and PPAR*γ*, encoded by separate genes, are present in humans [13], and each shows a distinct structure and function [9]. Although each is found ubiquitously throughout the body, expression levels can vary widely between tissues and the pattern is unique to each receptor type [14–16]. The role of PPAR*γ* in lung cancer biology is better understood and its potential role as a tumor suppressor is better characterized than those of PPAR*α* or PPAR*β*/*δ* [17].

PPAR*γ* is transcribed from one of three mRNAs—*γ*1, *γ*2, and *γ*3—that differ in sites of transcriptional initiation and splicing [18–20]. PPAR*γ*1 mRNA is found ubiquitously in humans [18], but expression of PPAR*γ*2 and PPAR*γ*3 mRNAs is more restricted [13]. Interestingly, *γ*1 and *γ*3 mRNAs translate into indistinguishable proteins [20], resulting in detection of only two protein isoforms—PPAR*γ*1 and PPAR*γ*2—in humans, with PPAR*γ*1 being more common than PPAR*γ*2 [19]. PPAR*γ*, initially known as a key regulator of adipocyte differentiation and glucose and lipid homeostasis [9, 18], has now demonstrated its involvement in the biology of lung cancer, as discussed in detail below, as well as in a wide variety of other cancers [17].

Both SCLC and NSCLC express PPAR*γ* [21–25]. However, the receptor is believed to be inactive in lung cancer cells, as suggested by cytoplasmic accumulation that reflects activation failure, perhaps due to some modification in its functional domains or to the absence of ligands in these cells [26]. Indeed, somatic loss-of-function mutations in the PPAR*γ* gene have been identified and implicated in the sporadic development of colorectal carcinomas; these mutations were shown to result in defective ligand binding and/or transcriptional activity [27]. An immunohistological analysis of 147 primary NSCLC tumor specimens also revealed that PPAR*γ* expression correlates with histological type and grade [10, 28]; well-differentiated adenocarcinoma shows higher PPAR*γ* expression than poorly differentiated adenocarcinoma or squamous cell carcinoma. These studies provide evidence for the significance of PPAR*γ* in lung cancer.

A number of PPAR*γ* ligands, both natural and synthetic, have been identified. Naturally occurring molecules including saturated and unsaturated fatty acids, eicosanoid derivatives such as 15-deoxy-Δ^12,14^-prostaglandin J_2_ (15d-PGJ_2_), and nitrated fatty acids such as nitrated linoleic acid and nitrated oleic acid can activate PPAR*γ* [18, 29, 30]. Certain synthetic compounds, predominantly represented by thiazolidinediones (TZDs) such as pioglitazone, rosiglitazone, troglitazone, and ciglitazone, have also been recognized as potent PPAR*γ* agonists [18]. Binding of these agonists activates transcriptional activity of PPAR*γ* by displacing corepressors that maintain the receptor in its inactive state and allowing it to heterodimerize with retinoid X receptors and bind to specific peroxisome proliferator response elements in the promoter regions of its target genes [9]. Accumulating evidence demonstrates that PPAR*γ* agonists/activation are antitumorigenic in most cases, hindering proliferation, growth, and progression and inducing differentiation and apoptosis in lung cancer [15, 17, 23, 25]. The potential of PPAR*γ* agonists as a novel therapeutic approach in the treatment of lung cancer is thus compelling.

## 2. PPAR***γ*** in Lung Cancer: Multifaceted Effect

Manifold molecular mechanisms underlie the antitumor effects of PPAR*γ* and its activation in lung cancer. Its influence is exerted not only on cancer cells but also on the surrounding tumor microenvironment harboring noncancerous elements such as cells of the immune system, fibroblasts, adipocytes, and blood and lymphatic vessels [31]. In addition to the cellular components, the tumor microenvironment contains noncellular elements such as growth factors, cytokines, chemokines, and extracellular matrix (ECM) [31]. These surroundings have been shown to play a critical part throughout all phases of tumor development and progression via their interaction with cancer cells [31]. Research to date indicates that PPAR*γ* activation hinders tumor development and progression by modulating the differentiation, proliferation, apoptosis, and motility of cancer cells and by making the tumor microenvironment less hospitable for tumor growth and metastasis.

### 2.1. Effect on Tumorigenesis 

#### 2.1.1. Regulation of Cancer Cells

PPAR*γ* is a key regulator of cellular differentiation, a crucial factor in the receptor's antitumor potential. As noted, cells often dedifferentiate or elude terminal differentiation during carcinogenesis; accordingly, protein markers associated with cellular differentiation are downregulated in cancer cells. Gelsolin, an actin-binding protein, is one such marker; it is expressed at low levels in many cancers, including lung cancer, and becomes upregulated upon induction of* in vitro* differentiation [25]. PPAR*γ* activation by ciglitazone and 15d-PGJ_2_ in multiple NSCLC cell lines enhances expression of gelsolin, Mad, and p21, considered general differentiation markers, while reducing expression of lung lineage-specific markers associated with lung progenitor cells such as mucin 1 (MUC1) and surfactant protein-A (SP-A) [25]. Of note, ciglitazone treatment also promotes morphological changes in these cells that are consistent with more mature differentiation status [25]. Other research groups extended these findings by showing that such PPAR*γ* agonist-induced differentiation of A549 and NCI-H23 NSCLC cells reflects sustained extracellular signal-regulated kinase 1/2 (ERK1/2) activation that has been linked to cellular differentiation [21, 32]. PPAR*γ* activation also induces conversion of adenocarcinoma cells to a more differentiated phenotype with polarity, which is associated with normal epithelial cells [33]. These studies reinforce the antitumorigenic and prodifferentiation role of PPAR*γ* activation in lung cancer.

PPAR*γ* activation can also hamper cell proliferation and tumor growth and promote apoptosis. This antitumorigenic role has been demonstrated with a variety of PPAR*γ* ligands in various lung cancer cell lines as well as in mouse lung cancer models. Interestingly, multiple mechanisms, which may be partially dependent on cell type, are responsible for these antiproliferative and proapoptotic roles of PPAR*γ* agonists. Troglitazone inhibits cell growth and induces apoptosis of SQ-5 NSCLC cells by stimulating, in a PPAR*γ*-dependent manner, mRNA and protein expression of growth arrest and DNA damage-inducible protein 153 (GADD153), a transcription factor implicated in the promotion of apoptosis [34]. Similarly, ciglitazone and 15d-PGJ_2_ inhibit cell proliferation and promote apoptosis of H345 and H2081 SCLC cells and H1838 and H2106 NSCLC cells [35]. However, the underlying mechanism in these cells was found to be upregulation of p21 expression and downregulation of cyclin D1. Rosiglitazone suppresses cell proliferation in H1838, H1792, and A549 NSCLC cells by PPAR*γ*-mediated suppression of phosphoinositide 3-kinase (PI3K)/Akt signaling and stimulating phosphatase and tensin homolog deleted from chromosome 10 (PTEN) expression [36, 37]. Troglitazone can also exert antiproliferative effects by causing G_0_/G_1_ cell arrest in multiple NSCLC cell lines, as evidenced by the accumulation of cells in G_0_/G_1_ phase and decrease in the number of cells in S phase [21, 22, 32]. In A549 cells, cell cycle arrest is due to reduction in expression of two G_1_ phase regulators, cyclins D and E [21]. While the apoptotic pathway remains unaffected in A549 cells [21], troglitazone-mediated growth inhibition is a consequence of increases in caspases 3- and 9-dependent apoptosis in NCI-H23 NSCLC cells, as indicated by an increase in the number of cells in sub-G_1_ phase [32]. Investigation of signaling pathways involved in apoptosis of these cells revealed a reduction in B-cell lymphoma-2 (Bcl-2) and Bcl-w expression, sustained ERK1/2 and p38 activation, and a decrease in stress-activated protein kinase (SAPK)/c-Jun N-terminal kinase (JNK) expression [22]. Lastly, sulindac sulfide, belonging to another class of PPAR*γ* ligands (nonsteroidal anti-inflammatory drugs), has also been shown to decrease anchorage-independent growth, a common measure of tumorigenicity, in both NSCLC and SCLC cells [38]. Similar results were obtained using natural and synthetic PPAR*γ* ligands and PPAR*γ* overexpression [38].

These* in vitro* findings are supported by animal studies. Troglitazone and pioglitazone as well as sulindac sulfide significantly reduce primary tumor growth of A549 NSCLC cells in a xenograft mouse model [21, 38]. Moreover, in studies using mouse models of spontaneous lung adenocarcinoma, where mice are injected with tobacco carcinogens, rosiglitazone or pioglitazone treatment reduces the tumor burden and significantly delays disease progression [39–41]. This inhibition of tumor progression from hyperplasia to adenoma is a result of inhibited cell proliferation [39]. Similar results were seen in a carcinogen-induced spontaneous tumor model of lung squamous cell carcinoma [40]. Thus, prodifferentiation, antiproliferative, and proapoptotic functions of PPAR*γ* activation identify PPAR*γ* agonists as attractive agents for lung cancer.

#### 2.1.2. Regulation of the Tumor Microenvironment

Angiogenesis is a normal physiological process of new blood vessel formation that cancer cells often hijack for successful establishment of primary tumors and metastases [42]. New vasculature allows the tumor to grow beyond the limit otherwise imposed by passive oxygen and nutritional diffusion at both primary and secondary sites [42]. It also facilitates metastasis by providing easy access to the circulation that cancer cells must enter to reach secondary organs [43]. Under normal conditions, the process of angiogenesis is tightly regulated by a milieu of pro- and antiangiogenic factors; during tumorigenesis, however, the proangiogenic factors are favored, leading to continuous neovascularization [42]. These tumor-associated blood vessels are also highly permeable, unlike normal vasculature [42], further facilitating cancer cell trafficking and metastasis.

Vascular endothelial growth factor (VEGF) is a well-established proangiogenic factor [42]. In addition, members of the CXC chemokine family that contain an ELR motif, such as interleukin-8 (IL-8; CXCL8), epithelial neutrophil-activating protein 78 (ENA-78; CXCL5), and growth-regulated oncogene-*α* (GRO-*α*; CXCL1), can regulate angiogenesis by stimulating the chemotaxis of endothelial cells that form new blood vessels [44]. Rosiglitazone reduces VEGF secretion by Lewis lung carcinoma (LLC) cells and inhibits angiogenesis, thereby reducing the tumor burden in mice [45]. Likewise, by suppressing secretion of ELR-positive CXC chemokines from A549 cells and migration of endothelial cells, troglitazone and pioglitazone inhibit angiogenesis in a mouse xenograft model [46]. In addition to indirectly influencing angiogenesis through inhibition of proangiogenic factors, activation of PPAR*γ*, which is highly expressed in tumor-associated endothelial cells, can also block angiogenesis by directly suppressing endothelial cell growth [45]. Furthermore, 15d-PGJ_2_ has been shown to induce caspase-dependent apoptosis of endothelial cells [47], which is expected to have an antiangiogenic effect.

Stromal cells, especially myofibroblasts, within the tumor microenvironment are the major source of cytokines, growth factors, matrix metalloproteinases (MMP), and ECM proteins, providing significant support for tumor growth and progression [15, 48]. Transforming growth factor-*β* (TGF-*β*) signaling induces fibroblast differentiation into myofibroblasts [15], and its constitutive activity has been detected under pathological conditions in humans [48]. 15d-PGJ_2_, troglitazone, ciglitazone, and rosiglitazone suppress TGF-*β*-stimulated differentiation of human primary lung fibroblasts into myofibroblasts [49, 50], as does expression of constitutively active PPAR*γ* in IMR-90 human fetal lung fibroblast cells [50]. Treatment with PPAR*γ* agonists also inhibits type I collagen production by these human lung fibroblasts. TGF-*β*-induced fibronectin expression is likewise attenuated by the PPAR*γ* agonist pioglitazone in human mesangial cells [51]. This inhibition of fibronectin expression has been replicated in H1838 NSCLC cells treated with the PPAR*γ* ligands BRL49653, 15d-PGJ_2_, or troglitazone [52]. An increase in some ECM constituents, including fibronectin and type I collagen and consequent remodeling of the tumor microenvironment, contributes to the pathogenesis of cancer [53]. Thus, these antitumorigenic influences on the tumor microenvironment, combined with its antiangiogenic effect, further support PPAR*γ* agonists for the treatment of lung cancer.

### 2.2. Effect on Metastasis

The role of PPAR*γ* in lung cancer extends beyond the regulation of primary tumor formation; mounting evidence suggests that PPAR*γ* activation suppresses tumor metastasis. In a study using a mouse xenograft model, metastasis to the lungs of A549 cells implanted in the dorsal flank of the animals was significantly reduced in response to troglitazone or pioglitazone [21]. Moreover, the few metastases detected in the lungs of the PPAR*γ* agonist-treated animals were smaller and better circumscribed than those in the placebo-treated animals [21]. Similarly, a study using a rat orthotopic lung tumor model showed that PPAR*γ* overexpression abrogates the metastasis of H2122 cells injected into one lung to the contralateral lung (or to the mediastinum) by impairing the cells' invasive capability [33]. Rats harboring PPAR*γ*-overexpressing H2122 cells also survived longer than the control animals. A similar observation was made in a mouse xenograft model in which LLC cells were implanted subcutaneously into the dorsal region of the mice [45]; rosiglitazone treatment completely blocked lung metastasis, preserving the normal architecture of the lungs, while the lung tissue of the control mice was filled with metastatic cancer cells. Intriguingly, the authors of the study observed the presence of LLC cells within the lung blood vessels of the rosiglitazone-treated mice while detecting none in the lung parenchyma [45], implying that rosiglitazone inhibits metastasis by preventing cancer cells' extravasation from the circulation, an essential step during tumor metastasis [54].

The negative effect of PPAR*γ* activation on tumor metastasis is further supported by findings that ciglitazone significantly reduces activity and total expression of MMP-2 by NCI-H157 and H1299 NSCLC cells [25] and that rosiglitazone enhances the activity of tissue inhibitor of metalloproteinases, which essentially decreases the net proteolytic activity of MMPs [45]. MMPs are key regulators of ECM remodeling and disruption [55]. Furthermore, expression of certain MMPs is linked to the metastatic potential of tumors in mouse models and generally correlates with poor prognosis for human patients with different types of cancer [55], thus supporting the significance of PPAR*γ*-mediated MMP suppression in the context of tumor progression.

Acquisition of metastatic potential is often accompanied by alterations in expression of certain cell adhesion molecules. In some carcinomas, E-cadherin, which holds epithelial cell sheets together by intercellular attachments and maintains the cells in their quiescent state, becomes downregulated, while adhesion molecules associated with enhanced cell migration, such as N-cadherin, are upregulated [54]. This switch in cadherin expression is considered a hallmark of the physiological process designated epithelial-mesenchymal transition (EMT). Cancer cells' activation of this program promotes metastasis [54] through loss of intercellular adhesion with consequent dissemination, morphological changes that facilitate migration and invasion, secretion of proteolytic enzymes, and resistance to apoptosis.

TGF-*β* signaling can drive these cellular modifications, modulating a set of transcription factors such as Snail, Slug, Twist, and zinc finger E-box-binding homeobox 1/2 (ZEB1/2) [54], and the enhanced TGF-*β* expression in many malignancies, including lung cancer, is implicated in the metastatic potential of advanced-stage tumors [56]. TGF-*β* has been shown to induce EMT in A549 cells, manifested as phenotypic transformation from a cuboidal epithelial morphology to an elongated mesenchymal form as well as by loss of intercellular attachment [57]. This change in cell morphology is accompanied by the predicted shift in cadherin expression and enhanced migration and invasion capabilities. Troglitazone and rosiglitazone block TGF-*β*-induced EMT, preventing the downregulation of E-cadherin expression and the upregulation of N-cadherin and other mesenchymal cell markers [58]. The authors further characterized the anti-EMT effect of PPAR*γ* activation by showing that troglitazone and rosiglitazone reverse many of the alterations that A549 cells undergo during TGF-*β*-induced EMT; the two PPAR*γ* agonists maintain intercellular adhesion, suppress cell migration and invasion, and abrogate MMP-2 and MMP-9 secretion. The suppression of EMT by PPAR*γ* activation was found to be via inhibition of SMAD3 transcriptional activity, a downstream component of TGF-*β* signaling essential for the induction of EMT. As expected from their other data, these authors also found that lung metastasis of A549 cells injected into mice via tail vein is also reduced by troglitazone treatment.

Metastasis is a significant source of morbidity and mortality for patients with cancer, and more than half of lung cancer patients harbor distant metastases at the time of diagnosis [1]. Therefore, the ability of PPAR*γ* agonists to suppress the development not only of primary tumors but also of metastases makes their therapeutic application in lung cancer promising.

## 3. PPAR***γ*** Agonists for Lung Cancer: Clinical Evidence and Synergism with Established Therapy

The promising role of PPAR*γ* agonists as therapeutic agents for lung cancer, suggested by experimental studies, has also been documented by clinical data. A retrospective analysis of 87,678 diabetic male patients (11,289 TZD users and 76,389 nonusers) from 10 Veterans Affairs Medical Centers found that TZD use is associated with a 33% reduction in subsequent diagnosis of lung cancer, although no effect of TZD use was found on the incidence of colon or prostate cancer [59]. Moreover, risk reduction for lung cancer was significantly higher in African American TZD users. This is highly relevant as incidence and mortality rates of lung cancer are highest in non-Hispanic black males [1]. Of note, this analysis focused only on diabetic patients and excluded those with preexisting cancers. Thus, it remains to be determined whether this correlation applies to a nondiabetic population. To this end, a clinical trial (NCT00780234) that represents a more general population is currently underway to evaluate the effect of pioglitazone in the chemoprevention of lung cancer.

PPAR*γ* agonists also demonstrate a synergistic effect with traditional chemotherapeutic agents, enhancing their cytotoxic effect on cancer cells. Combining PPAR*γ* ligands such as rosiglitazone and GW1929 with the platinum-based drugs commonly used for lung cancer treatment, such as cisplatin and carboplatin, synergistically inhibits growth of multiple NSCLC cell lines [60]. The synergistic effect of combination therapy was also observed in a xenograft lung cancer model as well as a spontaneous colon cancer model, without additional toxicity to the animals. Investigation of the mechanism underlying synergy between these two classes of drugs revealed that PPAR*γ* activation reduces the expression of metallothioneins, known for their role in resistance to platinum-based drugs by protecting the cells from platinum toxicity. Similar* in vitro* and* in vivo* findings were reported by another research group using the PPAR*γ* agonists troglitazone and pioglitazone and the chemotherapeutic drugs cisplatin and paclitaxel [61]. The molecular mechanism of action was different between the two studies, however. In the latter study, the authors observed the benefit of the combination therapy only when chemotherapeutic agents were followed, rather than preceded, by PPAR*γ* agonist treatment and concluded that this phenomenon is probably due to the induction of PPAR*γ* expression in response to cisplatin and paclitaxel treatment [61].

Besides traditional cytotoxic chemotherapy, within the past 2 decades targeted therapies that attack specific molecules responsible for tumorigenesis have been gaining attention in the field [62]. The synergistic effect of PPAR*γ* agonists has been demonstrated with this new class of therapeutic agents as well. Rosiglitazone enhances the antiproliferative effect of gefitinib, an epidermal growth factor receptor inhibitor, in A549 cells [37]. A combination of troglitazone and lovastatin, an inhibitor of 3-hydroxy-3-methyl-glutaryl-coenzyme A reductase (HMG-CoA reductase), also shows significantly stronger suppression of CL1-0 lung adenocarcinoma cell growth than either drug alone [63].

## 4. Conclusions

Review of extensive experimental data combined with retrospective analysis of human data supports PPAR*γ* as a promising therapeutic target in cancer. In addition to demonstrating their effectiveness as monotherapy for lung cancer, PPAR*γ* agonists also show synergistic effects with standard chemotherapy and potentially prevent chemotherapeutic resistance. These attributes suggest that PPAR*γ* agonists, in combination with other therapies currently in use or in clinical trials, represent a novel, attractive approach in lung cancer therapy. Additional, well-designed clinical trials should be undertaken to test the efficacy and safety of these agents in the treatment of lung cancer.

## Abbreviations

15d-PGJ_2_: 15-Deoxy-Δ^12,14^-prostaglandin J_2_
Bcl-2: B-cell lymphoma-2
ECM: Extracellular matrix
EMT: Epithelial-mesenchymal transition
ENA-78: Epithelial neutrophil-activating protein 78
ERK1/2: Extracellular signal-regulated kinase 1/2
GADD153: Growth arrest and DNA damage-inducible protein 153
GRO-*α*: Growth-regulated oncogene-*α*
HMG-CoA reductase: 3-Hydroxy-3-methyl-glutaryl-coenzyme A reductase
IL-8: Interleukin-8
JNK: Jun N-terminal kinase
LLC: Lewis lung carcinoma
MMP: Matrix metalloproteinase
MUC1: Mucin 1
NSCLC: Non-small cell lung cancer
PI3K: Phosphoinositide 3-kinase
PPAR: Peroxisome proliferator-activated receptor
PTEN: Phosphatase and tensin homolog deleted from chromosome 10
SAPK: Stress-activated protein kinase
SCLC: Small cell lung cancer
SMAD3: SMAD family member 3
SP-A: Surfactant protein-A
TGF-*β*: Transforming growth factor-*β*
TZD: Thiazolidinedione
VEGF: Vascular endothelial growth factor
ZEB1/2: Zinc finger E-box-binding homeobox 1/2.
